# A case with multiple nodules and mucosal oedema of the trachea and both bronchi induced by IgG4-related disease

**DOI:** 10.1186/s12890-024-02926-w

**Published:** 2024-03-05

**Authors:** Atsushi Torii, Kahori Oshima, Akari Iwakoshi, Masahide Oki

**Affiliations:** 1 Department of Respiratory Medicine, National Hospital Organization Nagoya Medical Center, 4-1-1, Sannomaru, Naka-ku, 460-0001 Nagoya, Japan; 2 Department of Rheumatology, National Hospital Organization Nagoya Medical Center, Nagoya, Japan; 3 Department of Pathology, National Hospital Organization Nagoya Medical Center, Nagoya, Japan

**Keywords:** Airway stenosis, IgG4-related disease, Mucosal edema, Multiple nodules

## Abstract

**Background:**

IgG4-related disease is a systemic fibroinflammatory disease that is mainly seen in older men, and involves multiple organs, such as the pancreas and lungs. However, 75% of patients with IgG4-related lung disease are asymptomatic (if they are symptomatic, they mainly complain of nasal congestion, rhinorrhoea, chest pain, and cough) and are incidentally diagnosed through chest computed tomograph. Although, nodules in the airway and bronchial wall thickening are criteria for diagnosis, it is important that nodules have been reported in peripheral airways in several cases and rarely in the central airway.

**Case presentation:**

A 74-year-old woman previously diagnosed with Mikulicz’s disease presented with swelling of the eyelid margin on both sides and visual disturbances. Computed tomography revealed extensive multiple nodules and mucosal oedema of the trachea and both bronchi. On flexible bronchoscopy under local anaesthesia, extensive lesions were observed from the middle of the trachea to the carina, extending into both segmental bronchi. The nodules were continuous with the normal respiratory tract mucosa, and the surfaces were smooth with minimal neovascularisation. Due to the solid nature of the lesion, obtaining an adequate amount of specimen was challenging. Therefore, we used a 1.9 mm cryoprobe under intubation, resulting in minimal bleeding. Subsequently, the patient was diagnosed with IgG4-related lung disease.

**Conclusions:**

The present case is very rare because of the presence of multiple nodules, severe mucosal edema of the central airway and the absence of mediastinal lymphadenopathy, ground glass nodules, and lung masses. Therefore, it is important to consider differential diagnoses. Thus, we emphasise the importance of endobronchial cryobiopsy for obtaining an adequate number of tissue specimens in such cases to establish a definitive pathological diagnosis.

## Background

IgG4-related disease (IgG4-RD) is a systemic fibroinflammatory disease that is mainly seen in older men (male-to-female ratio of 3:1 in Japan), and involves multiple organs [1]. The most frequent complication is pancreatitis, with lung involvement occurring in approximately 20% of patients [2, 3]. However, 75% of patients with IgG4-related lung disease (IgG4-RLD) are asymptomatic and are incidentally diagnosed through chest computed tomography (CT) [1]. The main symptoms in symptomatic patients are nasal congestion, rhinorrhoea, chest pain, and cough (sometimes similar to asthma) [1, 4]. The most frequent lesion on chest CT is hilar mediastinal lymphadenopathy [2]. Additionally, multiple nodules in the lung, ground-glass opacities, thickening of the bronchial wall and bronchovascular bundles and pleural thickening or effusion are considered specific to the disease [2, 3]. Diffuse ground-glass opacities are often difficult to distinguish from nonspecific interstitial pneumonia and organizing pneumonia. Consequently, the distinguishing feature is the presence of extrathoracic lesions. In cases involving additional lesions, it is important to discuss the case with a multidisciplinary team [2]. Although, nodules in the airway and bronchial wall thickening are criteria for diagnosis, it is worth noting that nodules have been reported in peripheral airways in several cases and rarely in the central airway [4, 5].

## Case presentation

A 74-year-old woman experienced parotid gland enlargement and swelling of the eyelid margins on the left side. Following a biopsy, the patient was diagnosed with IgG4-related Mikulicz’s disease. Her symptoms improved spontaneously. Three years later, she presented with swelling of the eyelid margins on both sides accompanied by a visual disorder. The patient was referred to the departments of ophthalmology, rheumatology and, connective tissue disorders. CT which revealed extensive abnormalities in the trachea and both bronchi, was subsequently referred to our department.

**Fig. 1:**
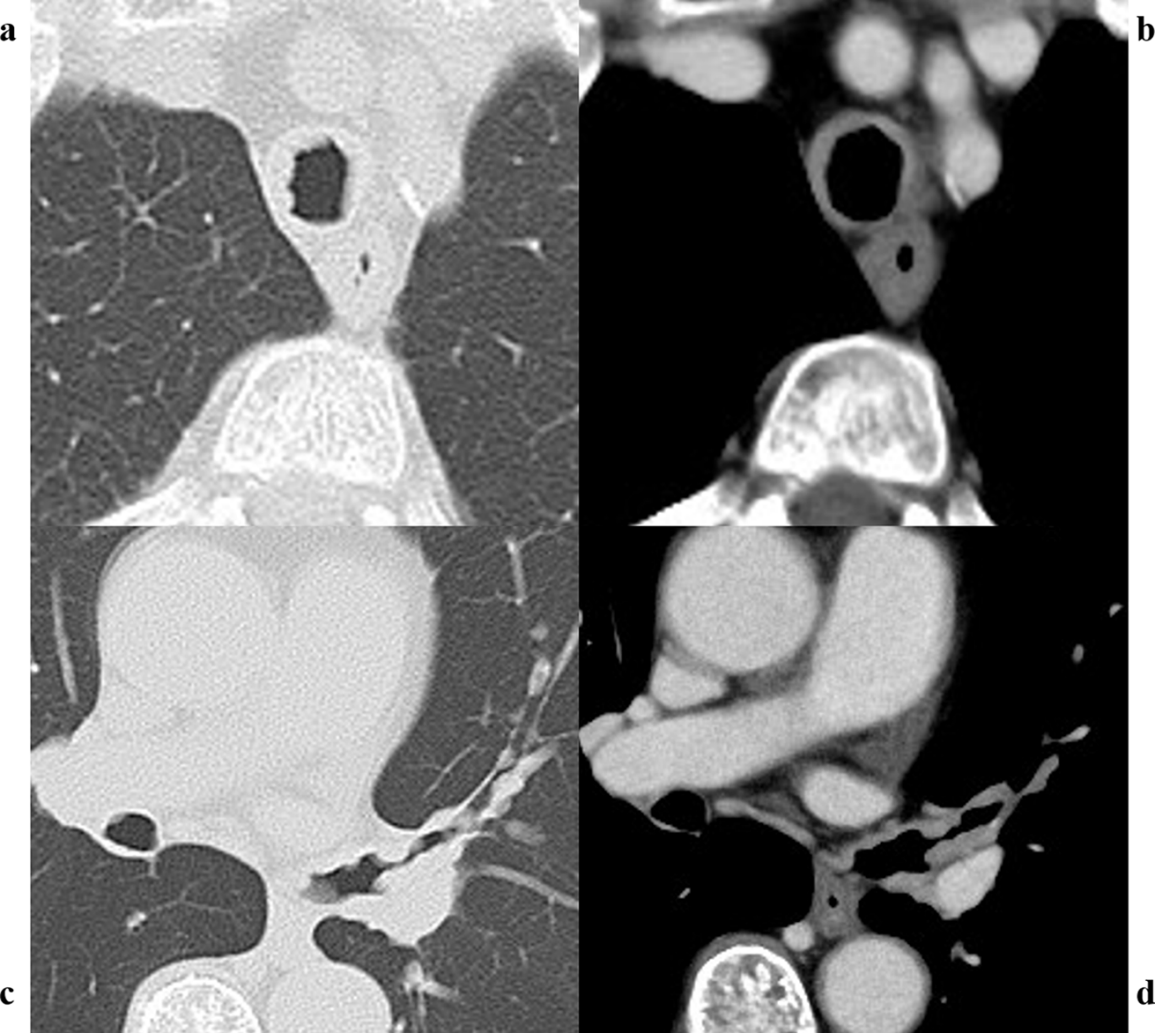
In the parenchyma (**a**) and mediastinal window settings (**b**), the tracheal wall appears to be thickened all around. Additionally, the bronchial wall is thickened and there are multiple nodules between the distal end of the left main bronchus and the left upper lobe bronchus (**c** and **d**)

**Fig. 2:**
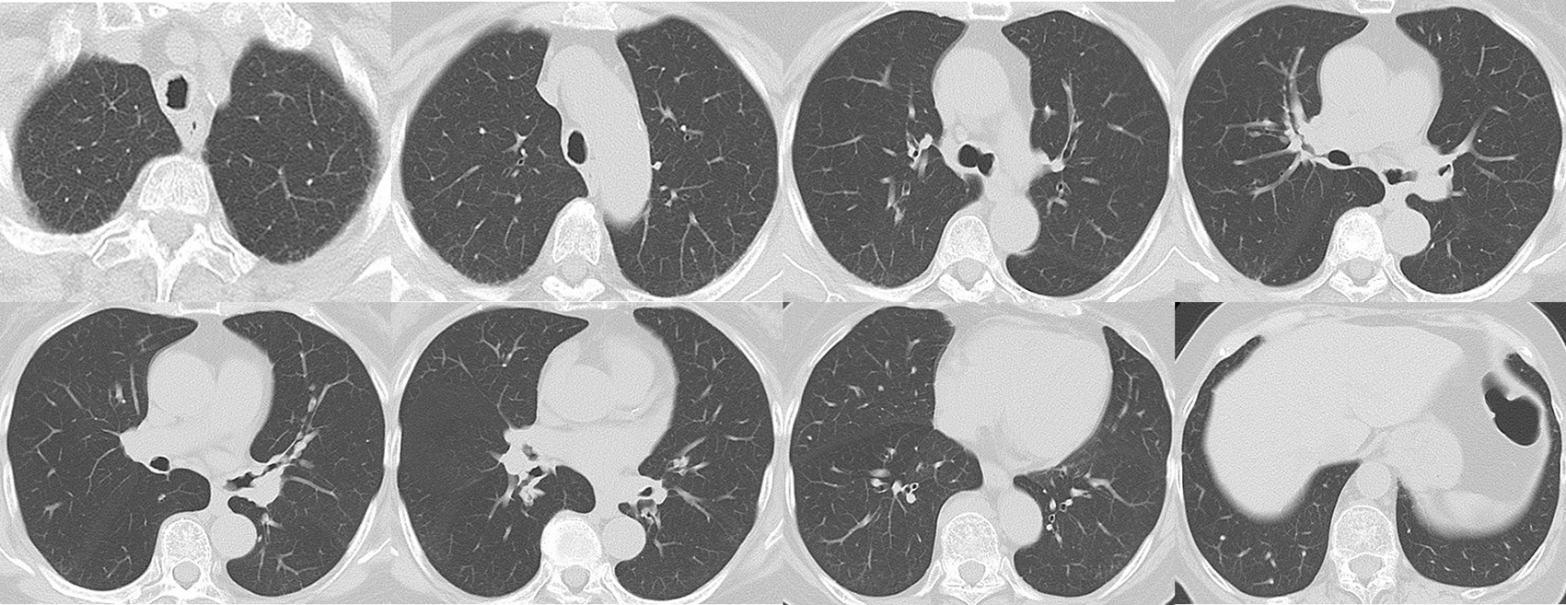
At the time of the initial diagnosis, in the parenchyma window settings, there were only thickening of bronchovascular bundles was observed, with no lung lesion such as ground-glass opacity, nodules, or interlobular septa

**Fig. 3:**
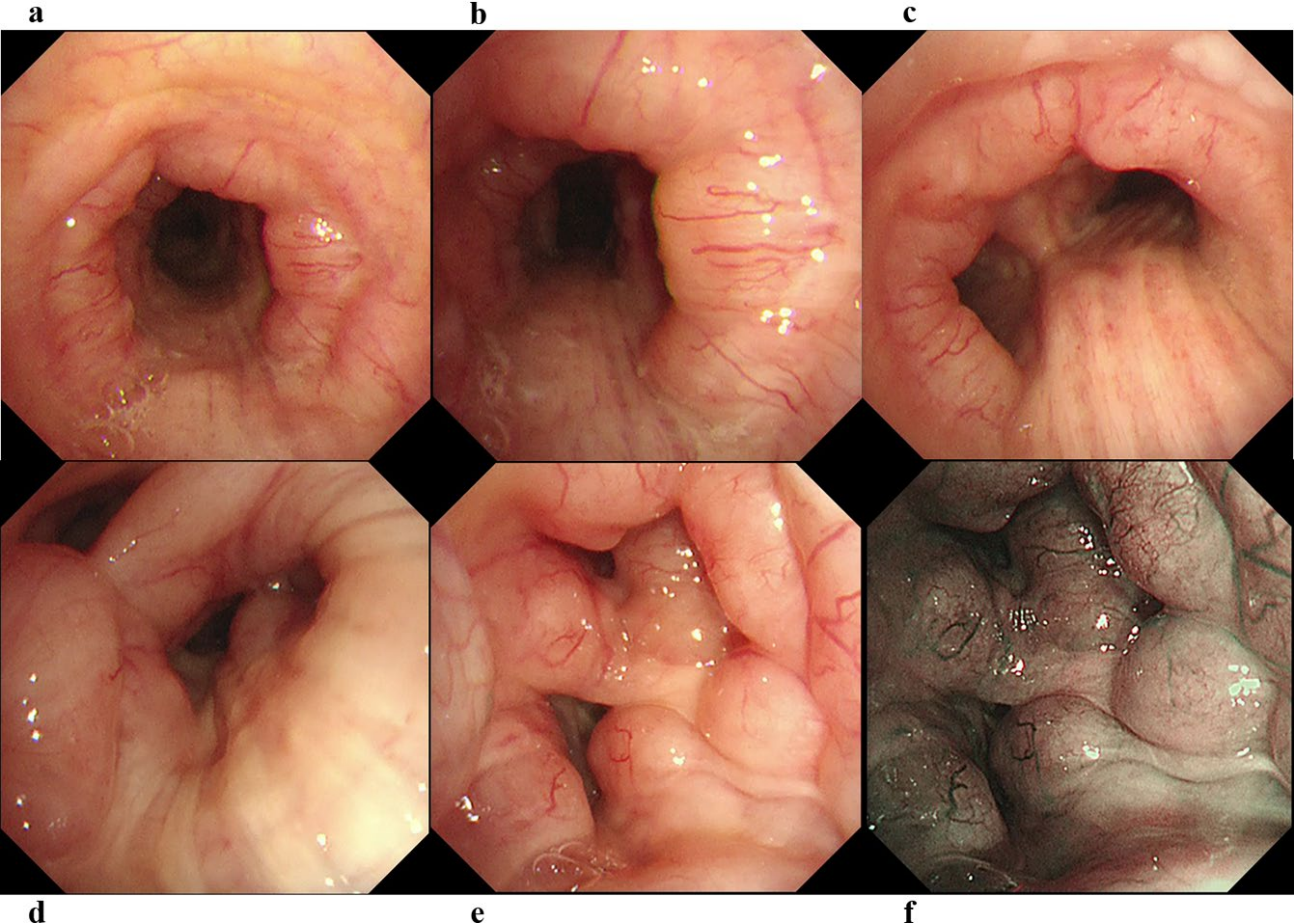
Bronchoscopy images show that the lesions extend from the middle trachea (**a**, **b**) to the carina (**c**) and from the bronchus intermedius (**d**) to the distal end of the left main bronchus (**e**). The stenosis was so severe that the Olympus 1TQ290 bronchoscope could not be passed through it. In the narrow band image (**f**), nodules and edema-like lesions can be seen, but the surface is smooth with little neovascularisation and continuity with the normal mucosa is maintained

**Fig. 4:**
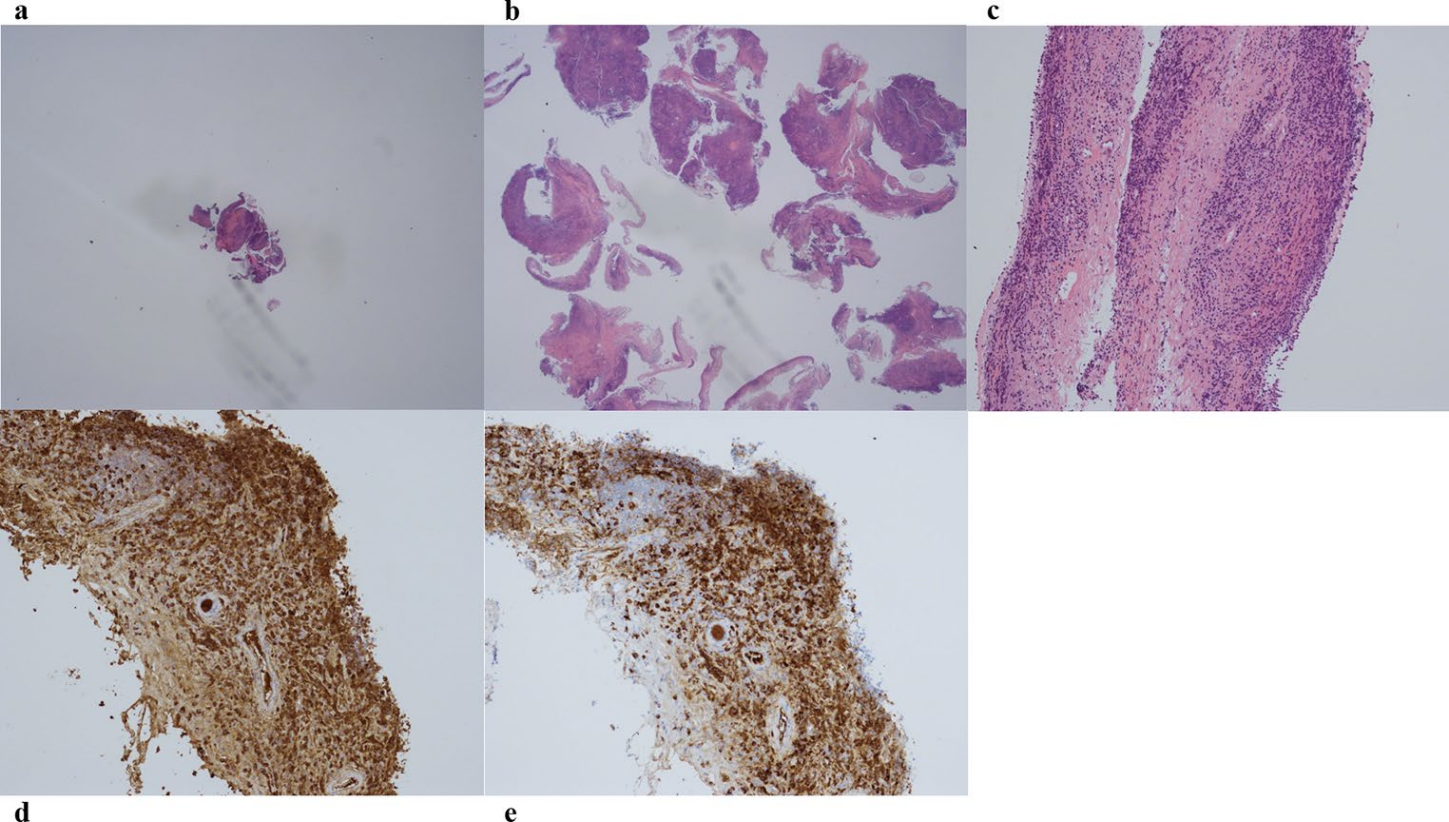
With standard forceps, a minimal amount of specimen is obtained (**a**, 2× object lens magnification, a total of 4 biopsies, 2.4mm^2^). In contrast, using a cryoprobe yields a sufficient amount of specimen for a definitive diagnosis (**b**, 2× object lens magnification, a total of 10 biopsies, 93.0mm^2^). Hematoxylin and eosin stained tissues show several invasive lymphocytes and plasma cells in the mucosa along with active fibrosis in a part of it (10× object lens magnification) (**c**). On IgG (**d**) and IgG4 (**e**) immunopathological staining, numerous positive plasma cells are visible with no difference among them. We detected > 10 IgG4 positive plasma cells per high power field and the IgG4 positive/IgG positive cell ratio was above 40% in every five areas

**Fig. 5:**
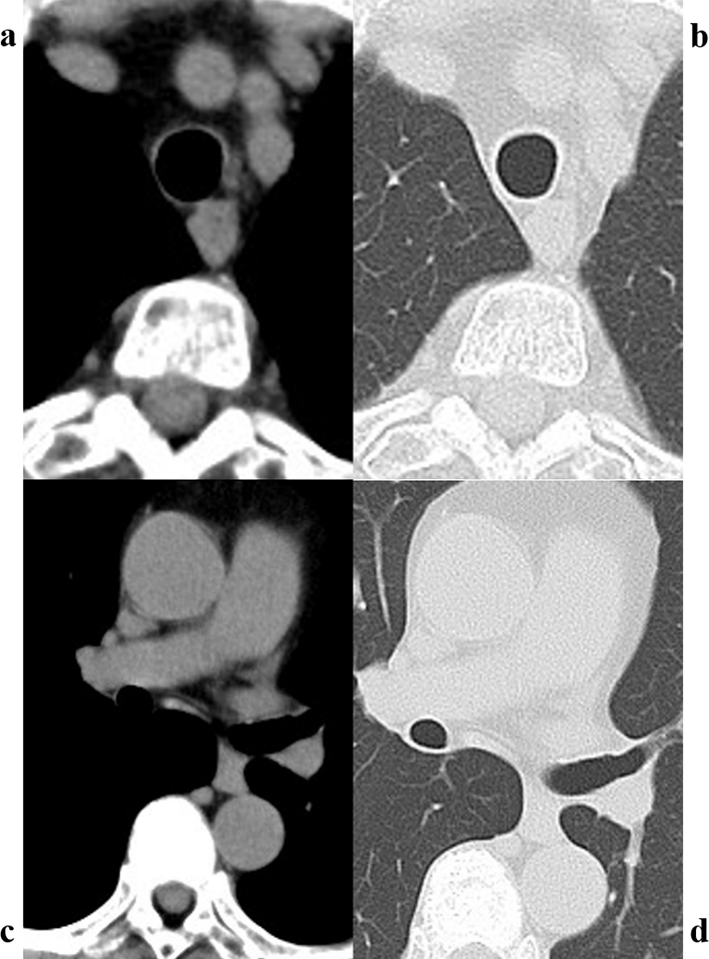
Chest computed tomography (CT) images at the level of the trachea (**a**, **b**) and the left main bronchus (**c**, **d**) after 6 months of treatment show considerable improvement in the lesions

**Fig. 6:**
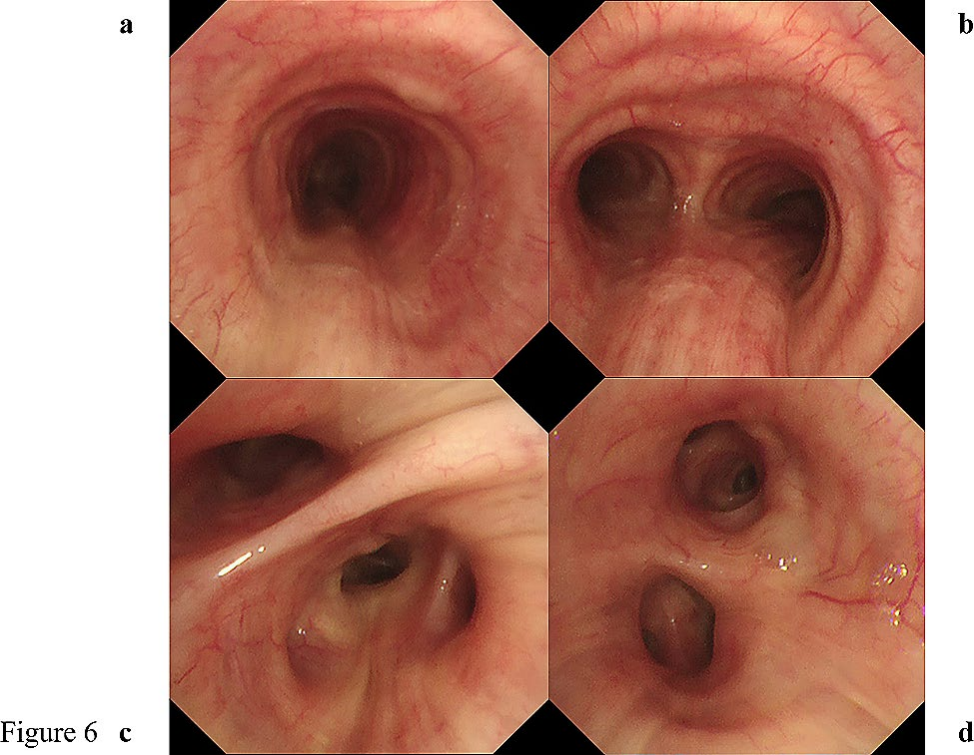
After 6 months of treatment, almost all lesions disappeared with only a few nodules visible at the 0 o’clock position in the trachea (**a**) and 6 o’clock position in the carina (**b**) on bronchoscopy. The right lower lobe bronchus (**c**) and the left main bronchus (**d**) exhibit a normal membrane

The patient’s vital signs were as follows: body temperature, 36.8 ℃; heart rate, 66 beats/min; blood pressure, 163/73 mmHg; and SpO_2_, 97% (on room air). Breath sounds were within the normal range. Routine blood investigation results were normal. Immunological examination results revealed the following: IgG, 3028 mg/dL (normal range: 861–1747); IgG4, 1994 mg/dL (normal range: 11–121); IgA, 220 mg/dL (normal range: 93–393); IgM, 39 mg/dL (normal range: 50–269); and IgE, 2143 IU/mL (normal range: 28–138). The angiotensin-converting enzyme level was 12.8 U/L, and IL-2R was 960 U/mL. Chest CT revealed extensive multiple nodules and mucosal oedema of the trachea and both bronchi; however, no ground-glass nodules or lung masses were observed (shown in Figs. 1 and 2). Pulmonary function tests revealed that the vital capacity (VC) was 2.02 L/min, %VC was 90.6%, forced expiratory volume in one second (FEV_1_) was 1.25 L, and FEV_1_% was 67.57%, peak expiratory flow volumes (PEF) was 2.13 L/s, suggesting an obstructive disorder. On flexible bronchoscopy under sedation, extensive lesions were observed from the middle of the trachea to the carina and further on both segmental bronchi. The nodules were continuous with the normal respiratory tract mucosa, and the surfaces were smooth with little neovascularisation. It was difficult to pass an Olympus BF-1TQ290 through the left carina 1 and the right carina 2 (shown in Fig. 3) because of the severe mucosal oedema. We tried to perform a biopsy using standard forceps, but the lesion was too solid to obtain an adequate amount of specimen (Fig. 4a). Subsequently, we used a 1.9 mm cryoprobe (ERBE; Medizintechnik, Tübingen, Germany) under intubation. Minimal bleeding was observed during the procedure. Following cryobiopsy (Fig. 4b), hematoxylin and eosin staining revealed a dense, non-monotonous lymphoplasmacytic infiltrate with focal interstitial fibrosis in the tracheal mucosa (shown in Fig. 4c). Immunostaining for IgG4 and IgG revealed abundant IgG4 + plasma cells, with > 40 IgG4 + plasma cells per high power field and an IgG4/IgG ratio of > 40% (shown in Fig. 4d, e); thus, these findings satisfied the diagnostic criteria for IgG4-RD. No malignant cells or areas were positive on Congo red staining. Consequently, the patient was diagnosed with IgG4-RLD. Treatment commenced with oral prednisolone at 30 mg/day (0.5 mg/kg/day). Subsequently, the symptoms of bilateral swelling of the lid margin and visual disorder rapidly improved. We continued prednisone at 30 mg/day for 4 weeks and then tapered it by 2.5-5 mg every 2–4 weeks [3, 6]. The symptoms did not relapse. The radiological findings on chest CT after 6 months of treatment are shown in Fig. 5. Bronchoscopy revealed only a few nodules (shown in Fig. 6). The pulmonary function test results also improved as follows: VC, 2.43 L; %VC, 110.0%; FEV_1_, 1.79 L; FEV_1_%, 69.38%; and PEF 4.16 L/s.

## Discussion and conclusions

The major differential clinical diagnoses of IgG4-RD are multicentric Castleman’s disease, anti-neutrophil cytoplasmic antibody-associated vasculitis (especially granulomatosis with polyangiitis and eosinophilic granulomatosis with polyangiitis), sarcoidosis, interstitial lung disease, lung cancer, and Rosai-Dorfman disease [1–3, 7]. The diagnostic criteria for IgG4-RLD are presented in Table 1 [2]. Therefore, the elevation of serum IgG4 is not considered specific enough.

The present case is considered rare due to the presence of multiple nodules and mucosal edema of the central airway, and the absence of mediastinal lymphadenopathy, ground-glass nodules, and masses in the lungs [4, 5]. Therefore, it is classified as dissimilar to the bronchovascular type [8]. Therefore, other differential diagnoses, such as amyloidosis [9], mucosa-associated lymphoid tissue lymphoma [10], inflammatory myofibroblastic tumour [11], and adenoid cystic carcinoma [12] should be considered. Thus, to establish a pathological diagnosis, it is crucial to perform a transbronchial biopsy, aiming to obtain a larger specimen size. We can obtain a larger specimen size using a cryoprobe compared to standard forceps [13], and a longer freezing time is associated with a larger specimen size [14]. In this case, the pathological findings satisfied the definitive diagnosis criteria described above by endobronchial cryobiopsy. Therefore, we believe that performing endobronchial cryobiopsy is suitable for obtaining large specimens and samples in cases that are too solid to perform a standard biopsy. If there are not enough devices for cryobiopsy, we consider it another option to perform rigid forceps biopsies during rigid bronchoscopy.

In conclusion, the presence of multiple nodules and mucosal oedema around the trachea and central bronchi is a rare occurrence in IgG4-RD. Therefore, in the differential diagnosis of IgG4-RD, it is crucial to consider not only benign diseases but also malignant tumours. It is important to perform transbronchial biopsy to obtain sufficient tissue samples for a definitive diagnosis, and thus, cryobiopsy may be a preferable method for this purpose.

**Table 1:** Diagnostic criteria for IgG4 related lung disease

1. Abnormal shadow on chest CT
・Hilar/mediastinal lymphadenopaty
・Thickening of bronchial wall, bronchovascular bundle, interlobular septal wall
・Nodular shadow, infiltrative shadow, pleural thickening/effusion
2. Elevated serum IgG4 concentration (> 135 mg/dL)
3. Pathological findings satisfing the following two items or more: a: >three items, b: two items
1) Dense lymphoplasmacytic cell infiltration into respiratory organ tissues
2) IgG4+/IgG + cell ratio > 40% and/or > 10 IgG4 + cells/high power field
3) Obliterative phlebitis or obliterative arteritis
4) Characteristics fibrosis, typically storiform pattern
4. Presence of lesions in the extrathoracic organs satisfying the diagnostic criteria of IgG4-related disease
<reference finding > hypocomplemenemia

Definite diagnosis satisfies 1 + 2 + 3a or 1 + 2 + 3b + 4, probable diagnosis satisfies 1 + 2 + 4 or 1 + 2 + 3b + reference findings, and possible diagnosis satisfies 1 + 2 + 3b

## Data Availability

The datasets used and analysed during the current study are available from the corresponding author on reasonable request.

## Abbreviations

CT: computed tomography
FEV_1_: forced expiratory volume in one second
IgG4-RD: IgG4-related disease
IgG4-RLD: IgG4-related lung disease
PEF: peak expiratory flow volume
VC: vital capacity
